# Characterization of pediatric cystic fibrosis airway epithelial cell cultures at the air-liquid interface obtained by non-invasive nasal cytology brush sampling

**DOI:** 10.1186/s12931-017-0706-7

**Published:** 2017-12-28

**Authors:** Aline Schögler, Fabian Blank, Melanie Brügger, Seraina Beyeler, Stefan A. Tschanz, Nicolas Regamey, Carmen Casaulta, Thomas Geiser, Marco P. Alves

**Affiliations:** 1 Department of Clinical Research, University of Bern, Bern, Switzerland; 2 Division of Respiratory Medicine, University Children’s Hospital of Bern, Bern, Switzerland; 3 Department of Pulmonary Medicine, University Hospital of Bern, Bern, Switzerland; 4 Graduate School for Cellular and Biomedical Sciences, University of Bern, Bern, Switzerland; 5 Institute of Virology and Immunology, Federal Department of Home Affairs, Mittelhäusern, Switzerland; 6 Department of Infectious Diseases and Pathobiology, Vetsuisse Faculty, University of Bern, Bern, Switzerland; 7 Institute of Anatomy, University of Bern, Bern, Switzerland; 8 Lucerne Children’s Hospital, Lucerne, Switzerland

**Keywords:** Cystic fibrosis, Pediatric, Air-liquid interface, Airway epithelium, Cytology brush, Nasal brushing

## Abstract

**Background:**

In vitro systems of primary cystic fibrosis (CF) airway epithelial cells are an important tool to study molecular and functional features of the native respiratory epithelium. However, undifferentiated CF airway cell cultures grown under submerged conditions do not appropriately represent the physiological situation. A more advanced CF cell culture system based on airway epithelial cells grown at the air-liquid interface (ALI) recapitulates most of the in vivo-like properties but requires the use of invasive sampling methods. In this study, we describe a detailed characterization of fully differentiated primary CF airway epithelial cells obtained by non-invasive nasal brushing of pediatric patients.

**Methods:**

Differentiated cell cultures were evaluated with immunolabelling of markers for ciliated, mucus-secreting and basal cells, and tight junction and CFTR proteins. Epithelial morphology and ultrastructure was examined by histology and transmission electron microscopy. Ciliary beat frequency was investigated by a video-microscopy approach and trans-epithelial electrical resistance was assessed with an epithelial Volt-Ohm meter system. Finally, epithelial permeability was analysed by using a cell layer integrity test and baseline cytokine levels where measured by an enzyme-linked immunosorbent assay.

**Results:**

Pediatric CF nasal cultures grown at the ALI showed a differentiation into a pseudostratified epithelium with a mucociliary phenotype. Also, immunofluorescence analysis revealed the presence of ciliated, mucus-secreting and basal cells and tight junctions. CFTR protein expression was observed in CF (F508del/F508del) and healthy cultures and baseline interleukin (IL)-8 and IL-6 release were similar in control and CF ALI cultures. The ciliary beat frequency was 9.67 Hz and the differentiated pediatric CF epithelium was found to be functionally tight.

**Conclusion:**

In summary, primary pediatric CF nasal epithelial cell cultures grown at the ALI showed full differentiation into ciliated, mucus-producing and basal cells, which adequately reflect the in vivo properties of the human respiratory epithelium.

**Electronic supplementary material:**

The online version of this article (10.1186/s12931-017-0706-7) contains supplementary material, which is available to authorized users.

## Background

Ciliated cells, secretory cells and basal cells are considered the most abundant cell types found in the epithelium of the human airways. The tight junctions between epithelial cells preserve the epithelial integrity. In addition, pathogens and particulate matter can be trapped in the secreted mucus and transported out of the airways by the coordinated beating of ciliated cells, also known as the mucociliary escalator [1, 2]. In vitro models based on primary airway epithelial cells are important tools to study molecular and functional aspects of the human respiratory epithelium. However, many researchers still rely on cell lines or undifferentiated airway epithelial cell cultures grown under submerged culture conditions as monolayers, which do not appropriately represent the situation found in vivo. In a more advanced in vitro cell culture model, airway epithelial cells are grown at the air-liquid interface (ALI) and re-differentiated into a ciliated pseudostratified epithelium covered by a layer of mucus. This system of a differentiated mucociliary epithelium could therefore serve as a preferable cell culture model that resembles more closely the in vivo properties of the native respiratory epithelium in structure (highly motile cilia and tight junction formation) and function (transepithelial electrical resistance and mucus production/secretion/transport) [3]. Whereas ALI cultures based on healthy airway epithelial cells are widely used, a limited number of investigators are working with cell cultures grown at the ALI in the field of CF research, and these fully differentiated CF airway epithelial cell cultures have so far been only partially characterized [4–6]. In addition, the majority of studies available in the literature using CF bronchial or nasal ALI cultures are based on CF cell lines or primary cultures obtained by the mean of surgery-based invasive methods from adult patients [7–10]. Therefore, we aimed at characterizing further fully differentiated pediatric CF airway cell cultures grown at the ALI by using nasal cytology brush sampling. Since it is ethically and technically easier to isolate primary nasal compared to bronchial epithelial cells, there is an interest in using nasal cultures as a surrogate of bronchial cells for studies based on airway epithelial cells [11–14].

## Methods

### Study participants

Fifteen pediatric CF patients were recruited at the Children’s University Hospitals of Bern. The clinical characteristics of the CF patients are shown in Table 1. Seven healthy human volunteers were recruited with the following exclusion criteria: recurrent nasal bleeding, therapy with anticoagulants, and a current respiratory tract infection. The ethics committee of the Canton of Bern, Switzerland approved the study (authorization KEK/09) and informed consent was obtained from all patients/volunteers.

**Table 1:** Demographic and clinical characteristics of study participants

Patient	Sex	Age (years)	Atopy (yes/no)^a^	Steroid use^b^	FEV1%	*P. aeruginosa* (yes/no)^c^	Genotype	AZM long-term (yes/no)
1	f	2.6	no	no	ND	no	F508del/F508del	no
2	m	3.0	no	no	ND	yes	F508del/F508del	no
3	f	4.5	no	no	ND	no	F508del/F508del	ND
4	f	15.2	yes	no	58	yes	F508del/I507del	no
5	f	5.6	no	no	68	yes	F508del/F508del	no
6	m	13.2	yes	no	57	no	F508del/F508del	no
7	m	3.7	no	no	ND	no	F508del/F508del	no
8	f	7.7	no	no	70	yes	F508del/3905insT	yes
9	m	2.6	no	no	ND	no	F508del/F508del	no
10	m	12.7	no	yes	86	no	F508del/F508del	no

^a^defined as positive history of hayfever, eczema or asthma

^b^defined as any treatment with systemic, inhaled or nasal steroids within the past 3 months

^c^defined as at least one P. aeruginosa-positive oropharyngeal culture during the preceding 12 months

*abreviation: ND* not determined

### Cell culture

Primary nasal epithelial cells were obtained by nasal brushing as previously described [11, 15, 16]. Briefly, the nasal epithelial cells were obtained by brushing the inferior surface of the middle turbinate of both nostrils twice by using cytology brushes (Dent-o-care, London, UK). Next, the freshly brushed tissue was seeded in collagen-coated (Advanced BioMatrix Inc., San Diego, CA, USA) 12.5 cm^2^ cell culture flasks (BD Bioscience, USA) in Bronchial Epithelial Growth Medium (BEGM, Lonza, Switzerland) supplemented with Single Quots (Lonza, Switzerland) and Primocin (100 μg/ml, InvivoGen, US) in a humidified incubator at 37 °C. The CF cells were additionally treated with Amphotericin B (250 μg/ml; Sigma Aldrich, US) and Ceftazidime (100 μg/ml, GlaxoSmithKline, Switzerland) during five days after sampling [16]. Out of 15 CF patients brushed, 5 cultures were lost due to poor cell growth during the expansion phase of the cultures. The 10 CF patients from which successful ALI cultures could be established and used for further investigation are presented in Table 1. We obtained 0.4 to 1.5 million viable cells per CF patient after brushing and the time to reach confluence during the expansion phase was 7 to 15 days. The time to confluence during the expansion phase was directly dependent on the number of cells obtained after brushing. The success rate of culture establishment for healthy donors was higher compare to CF patients (7 healthy donors brushed resulted in 6 cultures successfully established). Of note, when growing on the inserts, we obtained a 100% success in differentiation of the cultures. Once confluent under submerged condition, the cells where seeded at a density of 60,000 cells per insert onto 24-well inserts with a pore size of 0.4 μm at 37 °C, 5% CO_2_ (Greiner Bio-One, Austria). Cells were grown on the insert membranes under submerged conditions by adding 200 μl of BEGM apically and 450 μl of BEGM in the basal chamber until they reached confluence (2–3 days post seeding). Cell cultures were then washed with phosphate buffered saline (PBS) 1X w/o Ca^2+^ and Mg^2+^ and culture medium was then changed to complete PneumaCult™-ALI Medium (Stemcell Technologies, CA) following the manufacturer’s instruction. Briefly, cell cultures were exposed to air on the apical side and provided with 450 μl of complete PneumaCult™-ALI Medium in the basal compartment to promote mucociliary differentiation. The basal medium was changed every second day. Twenty-one to 28 days post exposing the cells to air at 37 °C, 5% CO_2_, fully differentiated primary CF airway epithelial cell cultures were obtained and showed evidence of mucus production and ciliary beating. According to the supplier’s specifications, the differentiated CF nasal epithelial cells were therefore used at 21 to 28 days post exposure to air for further experiments. The control bronchial ALI cultures form 2 different donors used for the evaluation of CFTR expression by confocal microscopy were obtained commercially (Epithelix Sàrl, Geneva, Switzerland).

### Histology

Cell culture membranes were cut from the insert and fixed for 20 min in 70% ethanol. Samples were then stained with hematoxylin and eosin (H&E) and snap-frozen in Tissue-Tek O.C.T. Compound (Sakura Finetek USA Inc., US). Cryosections of 7 μm were cut (30,505 Cryostat, Leica Microsystems, Heerbrugg, Switzerland) and examined by brightfield microscopy.

### Immunofluorescence

Cells on membrane cut from the insert were fixed in 70% ethanol for 20 min and were permeabilized with 0.2% Triton X-100 (Sigma-Aldrich, US) for 15 min and washed with staining buffer (PBS containing 0.1% BSA and 0.001% NaN_3_ (Sigma Aldrich, US) for 5 min at room temperature. The cells were then incubated with the primary antibody or dye in staining buffer for one hour at room temperature. Cells were stained with Phalloidin Rhodamine (1:100; Invitrogen, Switzerland), rabbit anti-MUC5AC (clone H-160, 1:100, Santa Cruz Biotechnologies, US) mouse anti-β-tubulin (clone 2 28 33, 1:100, Invitrogen, Switzerland), rabbit anti-p63 (clone EPR5701, 1:60, Abcam, UK), rabbit anti-ZO-1 (Zona occludens-1; ab59720, 1:50, Abcam, UK), and mouse anti-CFTR (clone 24–1, 1:100, R&D Systems, UK). After washing with staining buffer, the cells were incubated for one hour with the secondary antibody in staining buffer at room temperature: anti-mouse Alexa Fluor 488 (Invitrogen, US) and anti-rabbit Alexa Fluor 647 (Invitrogen, US). Cells were washed with staining buffer and then mounted in DAPI mounting media (4′,6-diamidino-2-phenylindole; Vector Laboratories Inc., US). A Zeiss Laser scanning microscope (LSM) 710 (Carl Zeiss AG, Switzerland) was used to take the optical sections and image processing was done using Imaris software (Bitplane AG, Switzerland).

### Transmission electron microscopy

Membranes with fully differentiated CF nasal epithelial cells were cut from inserts and were fixed in buffered 2.5% glutaraldehyde (Agar Scientific Ltd., Plano GmbH, Wetzlar, Germany) and post-fixed in buffered 1.0% osmium tetroxide (SPI Supplies, West Chester, USA). Preparations were then dehydrated in graded ethanol (Alcosuisse, Switzerland) and embedded in Epon (Fluka, Buchs, Switzerland) and left to harden at 60 °C for 5 days. Ultrathin sections (70–80 nm) were produced using an ultramicrotome UC6 (Leica Microsystems, Vienna, Austria) and mounted on single slot copper grids and subsequently stained with uranyl acetate and lead citrate utilizing an ultrostainer (Leica Microsystems, Vienna, Austria). Ultrathin sections were examined with a transmission electron microscope (TEM; CM12, Philips, Eindhoven, Netherlands).

### Ciliary beat frequency measurement

Membranes were cut from inserts and placed on a glass slide mounted with culture medium and covered with a coverslip. Good contrast region of the differentiated cell culture with beating cilia was first selected using the OLYMPUS Vanox-S microscope with SPLAN Apo objectives (10X, 20X, 40X) with differential interference contrast (DIC/Nomarski). By means of a Point Grey Flea 3 (FL3-U3-13Y3M-C) B/W high speed CMOS camera (Point Grey Research, Inc., Canada) that was connected to the microscopy system, image series of beating cilia were taken serially at 300 frames per second. To perform ciliary beat frequency, one ALI insert per CF patient was used and 20 different spots per insert where evaluated (10 near the border and 10 in the center). The measurement where performed under constant temperature of 30 °C and to minimize any bias, the measurements where performed in a minimal amount of time. Image processing was done using the Image J software (US) and ciliary beating frequency (CBF) was analyzed by means of the power spectrum based on Fast Fourier Transform algorithm.

### Cell layer integrity test

Cell layer integrity was measured in submerged cell cultures grown on inserts and in cell cultures grown on inserts at the ALI for one day to 24 days (fully differentiated) as follow: complete PneumaCult™-ALI Medium was removed from the basal chamber of CF nasal epithelial cells grown on 24-well inserts and cells were washed with PBS (Life Technologies, US). Two mg/ml of 4 kDa Fluorescein isothiocyanate-dextran (FD4, Sigma-Aldrich, US) was diluted in 150 μl complete PneumaCult™-ALI Medium and added apically to the cell cultures. The basal chamber was filled with fresh complete PneumaCult™-ALI Medium and cultures were then incubated for 4 h at 37 °C and 5% CO_2_. The fluorescence intensity of the contents of the basal chamber was determined with the following wavelengths: excitation 485 nm, emission 544 nm. Of note, a 24-well insert only was used as reference value and percentage of fluorescence was expressed relative to this control.

### Transepithelial electrical resistance

Fully differentiated CF nasal epithelial cell cultures were washed with PBS and fresh complete PneumaCult™-ALI Medium was added to the apical and basal chamber for equilibration for 30 min at room temperature. Trans-epithelial electrical resistance (TEER) was then assessed three times with an EVOM meter with the STX2 “chopstick” electrodes of 4 mm width and 1 mm thick (World Precision Instruments, FL) and a mean resistance was calculated. Values were then corrected for fluid resistance (insert with no cells) and surface area.

### Isolation of total RNA and RT-PCR

Total RNA harvesting was performed on inserts at the ALI for 24 days with the RNA II kit (Macherey-Nagel, Oensingen, Switzerland) and complementary deoxyribonucleic acid (cDNA) synthesis was done with Omniscript RT Kit (Qiagen, Valencia, CA, USA). RT-PCR measurements were performed with the Taqman Fast Universal PCR Master Mix (Applied Biosystems, Foster City, CA, USA) on a 7500 Fast Real-Time PCR System (Applied Biosystems). Sequences of the primers and probe used for the detection of the CFTR transcript: forward primer: 5’-AGCTGTCAAGCCGTGTTCTAGATA-3′, reverse primer 5’-ATGAGGAGTGCCACTTGCAAA-3′, probe 5’-FAM-CACACGAAATGTGCCAATGCAAGTCCTT-TAMRA-3′. The CFTR mRNA levels where standardized to the housekeeping ribosomal 18S RNA by using the following primers/probe set: forward primer 5’-CGCCGCTAGAGGTGAAATTCT-3′, reverse primer 5’-CATTCTTGGCAAATGCTTTCG-3′, probe 5’-FAM-ACCGGCGCAAGACGGACCAGA-TAMRA-3′.

### Enzyme-linked immunosorbent assay

Basolateral protein levels of interleukin (IL)-6 and IL-8 were measured with the DuoSet ELISA Development kit (R&D, Minneapolis, MN, USA). In our experience, more reproducible cytokine levels in the basolateral vs. apical compartment are obtained due to the difficult-to-standardize apical washing procedure. Also, this approach prevents the perturbation of the epithelium. In line with our experimental approach, sampling of the basolateral compartment for the evaluation of IL-6 and IL-8 levels produced by airway epithelial ALI cultures is often selected [6, 10, 17–19].

### Statistical analysis

Data are presented as mean ± SEM. An unpaired *t*-test was used to determine differences between two groups. To conduct the statistical analysis, GraphPad Prism 5 software (GraphPad Software Inc., US) was used. A *p* value <0.05 was considered statistically significant.

## Results

### Histological examination reveals a ciliated pseudostratified CF nasal epithelium

**Fig. 1:**
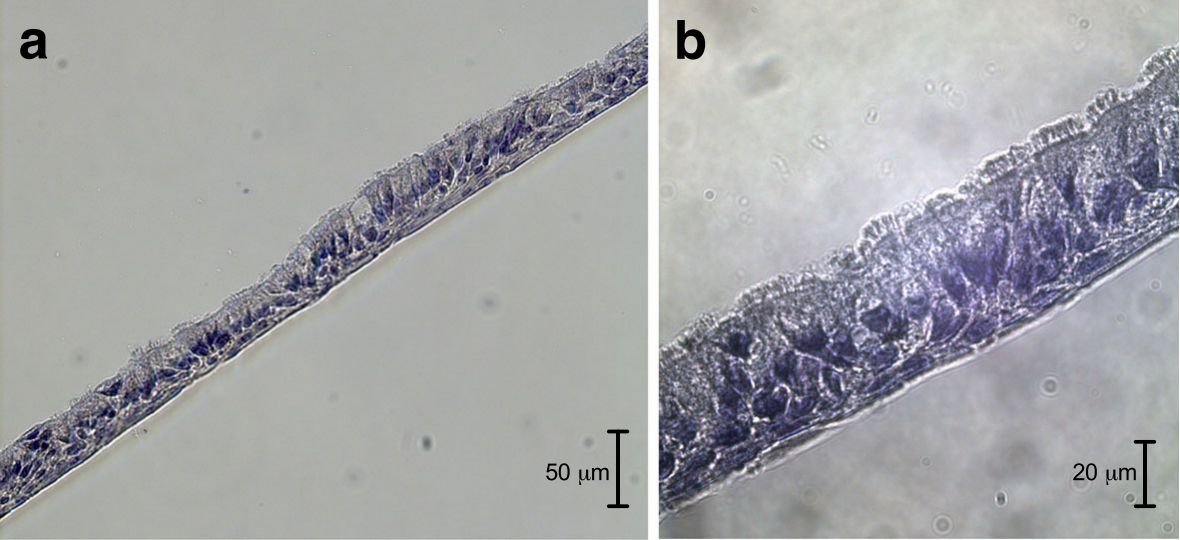
Histological morphology of differentiated cystic fibrosis cell cultures grown at the air-liquid interface. **a**, **b** Fully differentiated cystic fibrosis primary airway epithelial cell cultures were fixed in 70% ethanol for 20 min and embedded in Tissue-Tek O.C.T. Compound and subsequently stained with hematoxylin and eosin

Fully differentiated CF nasal epithelial cells (3 to 4 weeks post exposure to air) were fixed and stained with H&E to determine the structure of the differentiated epithelium. The stainings showed the structure of a pseudostratified epithelium with a ciliary brush border (Fig. 1a, b) indicative of a fully differentiated CF airway epithelium. Also, we noticed a productive secretion of mucus (see Additional file 1).

### Morphological characteristics of fully differentiated CF nasal epithelial cell cultures

**Fig. 2:**
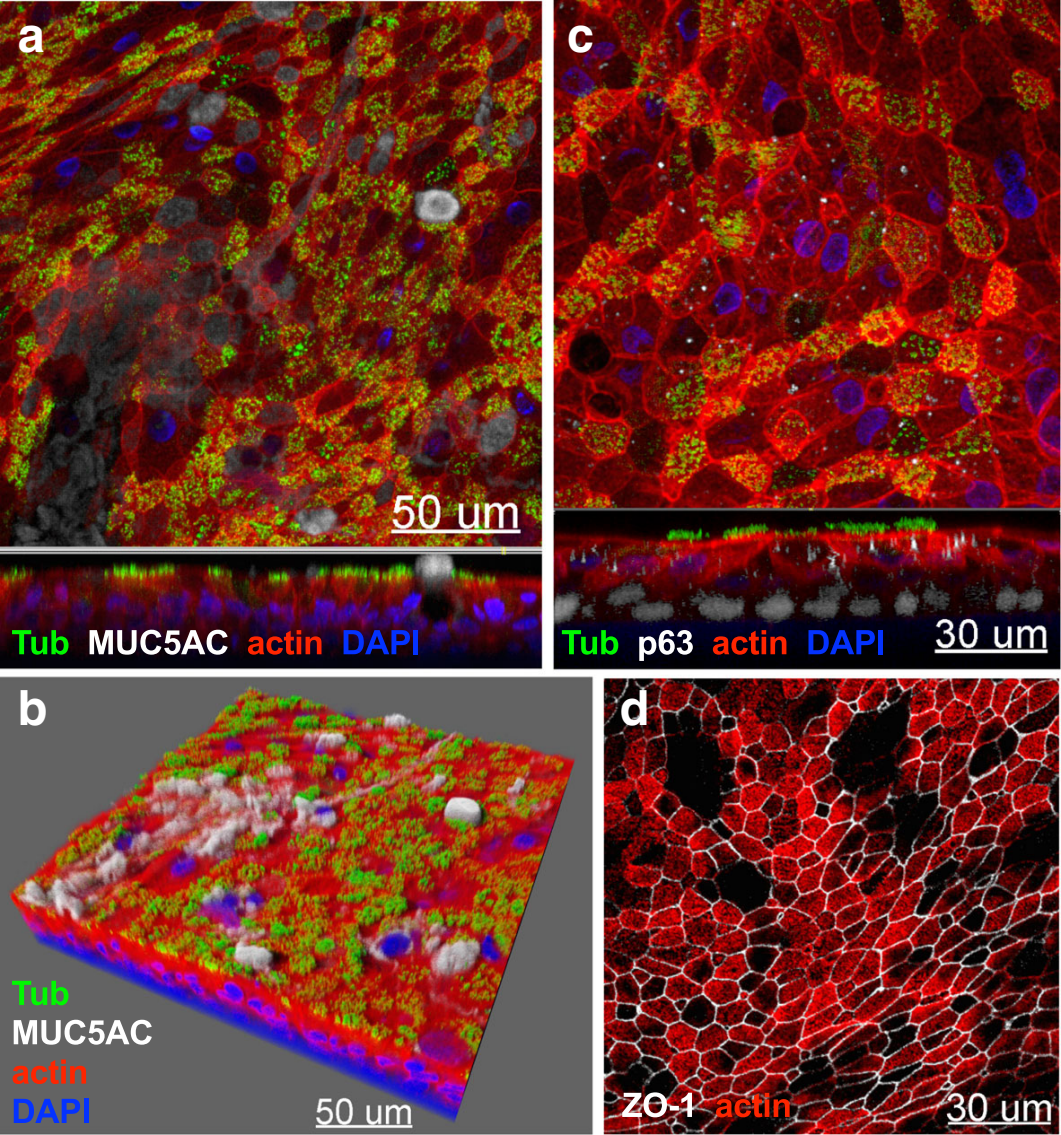
Differentiation of primary cystic fibrosis nasal epithelial cells into a mucociliary phenotype visualized by LSM. **a**, **b** Presence of β-tubulin-positive ciliated cells and MUC5AC-positive mucus-secreting cells at 4 weeks post exposure to air was shown by representative three-dimensional immunofluorescence stainings. xy-projection is shown on top panels and xz-projection is shown on lower panels. Blue: DAPI (nucleus); red: actin; green: β-tubulin; white: MUC5AC. **c** Presence of p63-positive basal cells at week 4 post exposure to air was shown by representative three-dimensional immunofluorescence stainings taken by LSM. xy-projection is shown on top panel and xz-projection is shown on lower panel. Blue: DAPI (nucleus); red: actin; green: β-tubulin; white: p63. **d** Immunofluorescence staining of the tight junctional protein ZO-1 at 4 weeks post exposure to ALI in the xy-projection. Red: actin; white: ZO-1. The micrographs presented are representative of data generated from six different CF patients

To investigate if the three main cell types of the native respiratory epithelium (ciliated, mucus-secreting and basal cells) were present in the differentiated CF nasal epithelial cell cultures, we analyzed immunofluorescence pictures at 3 to 4 weeks post exposure of cell cultures to air. The immunofluorescence pictures revealed fully differentiated CF cultures positive for ciliated cells (β-tubulin, green), mucus-secreting cells (MUC5AC, white) (Fig. 2a, b) and basal cells (p63, white) (Fig. 2c). Further a positive staining for ZO-1 (tight junction protein, white) suggested a tight, intact epithelial layer of the fully differentiated CF cell cultures (Fig. 2d).

### Electron microscope characterization of fully differentiated CF nasal epithelial cells

**Fig. 3:**
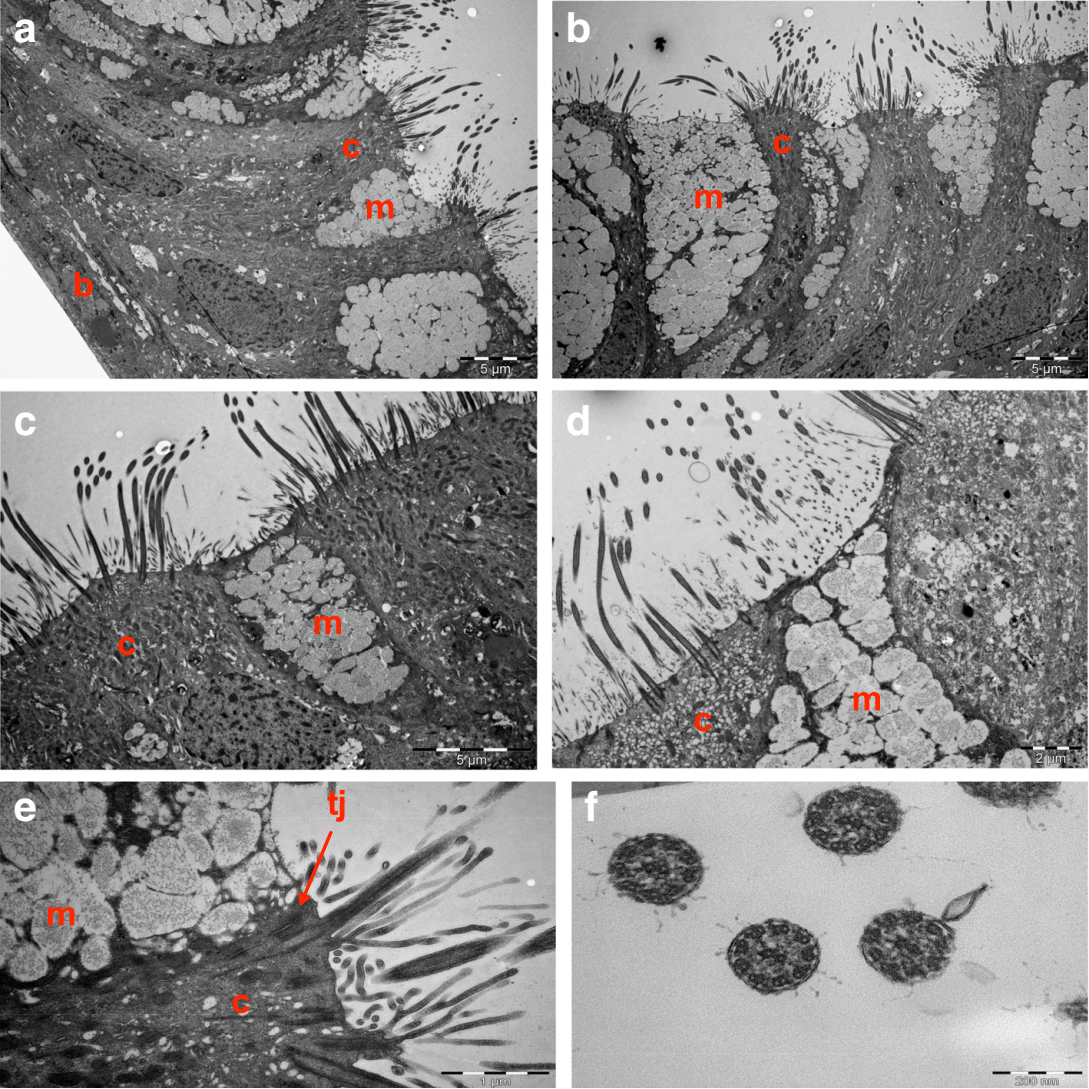
Electron microscopic characterization of differentiated cystic fibrosis cell cultures grown at the air-liquid interface. **a**-**d** Ciliated (*c*), mucus-secreting (*m*) and basal cells (*b*) revealed by electron micrographs of a primary culture of cystic fibrosis nasal epithelial cells grown at the air-liquid interface for 4 week. **e** Electron micrograph of tight junctions (*tj*) between a ciliated (*c*) and mucus-secreting (*m*) cell of a primary culture of cystic fibrosis nasal epithelial cells grown at the air-liquid interface for 4 weeks. **f** Electron micrograph of a primary culture of cystic fibrosis nasal epithelial cells grown at the air-liquid interface for 4 weeks showing the normal structure of cilia consisting of nine outer 18 microtubule doublets with dynein arms, surrounding one central pair of single microtubules. The micrographs presented are representative from cultures generated from two CF patients

Examination of the fully differentiated CF nasal epithelial cell cultures by TEM confirmed the pseudostratified organization of the epithelium and the mucociliary phenotype. Indeed, mucus-secreting cells with secretory granules and ciliated cells at the apical site and basal cells could be identified (Fig. 3a-d). Typical apical tight junctional complexes were observed between adjacent cells in the epithelium (Fig. 3e) and the cilia of ciliated cells showed the typical axonemal ultrastructure with its characteristic arrangement of microtubules and dynein arms (Fig. 3f).

### Ciliary beat frequency of fully differentiated CF nasal epithelial cell cultures

Evaluation of the CBF is an important and reliable method to assess the physiological condition of the respiratory epithelium. It is also an essential tool of clinical relevance in CF since the functional and coordinated ciliary beat is a pivotal component of the mucociliary escalator, which is involved in the development and progression of CF lung disease [20–22]. To investigate whether the ciliated cells showed a ciliary beat, the CBF of fully differentiated CF cell cultures established from three different CF patients was determined at 4 weeks post exposure to air. Three fully differentiated CF nasal epithelial cell cultures generated from 3 different CF patients revealed an average CBF of 9.67 ± 0.44 Hz. A recording of the ciliary beat of the CF cultures grown at the ALI is presented in Additional file 2.

### Epithelial barrier integrity of fully differentiated CF nasal epithelial cell cultures

**Fig. 4:**
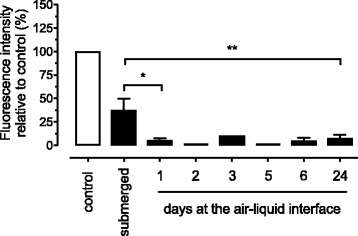
Epithelial tightness of fully differentiated CF cell cultures. Relative fluorescence intensity of FD-4 in the basal chamber was assessed at 4 h after apical addition of FD-4 on submerged and 1 to 24 days ALI cultures of primary CF nasal epithelial cells. Values were expressed as % of fluorescence relative to control (empty insert without cells and with medium only). Data are presented as mean of 1–10 different CF patients. Statistical analysis was done in comparison to submerged cultures. *: *p* < 0.05, **: *p* < 0.01

In order to determine if the cell layer of well differentiated CF cells was functionally tight, we performed an epithelial cell layer integrity test in submerged CF cultures and at 1–24 days post exposure to air by apically applying FD-4 on CF cultures and assessed the fluorescence intensity in the basal chamber. We found that the relative fluorescence intensity was significantly ca. 10 times lower already one day post exposure to air in comparison to submerged cultures, indicative of a tight epithelium (*p* < 0.05; Fig. 4). We performed preliminary measurements of TEER in fully differentiated cultures at week 4 post-exposure to air and found values in the range of other investigators such as Sajjan et al. at 500 Ωcm^2^ [4] and Wiszniewski et al. at 542 Ωcm^2^ [5], which is suggesting tight junction formation.

### Baseline CFTR and inflammatory cytokine levels of fully differentiated CF nasal epithelial cell cultures

**Fig. 5:**
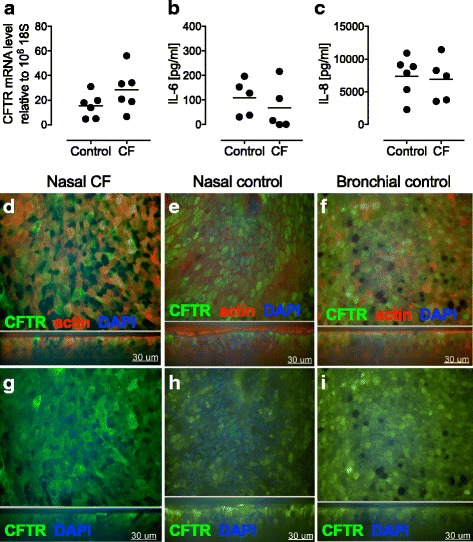
CFTR and baseline IL-6 and IL-8 levels in CF and healthy fully differentiated ALI cultures. CFTR mRNA levels (**a**) assessed by RT-PCR in fully differentiated CF and control ALI cultures. Baseline protein levels of IL-6 (**b**) and IL-8 (**c**) in the basolateral chamber of fully differentiated ALI cultures. Data are presented as mean of 5–6 patients/donors. CFTR staining in nasal CF (**d**, **g**) nasal healthy (**e**, **h**), and bronchial healthy (**f**, **i**) cultures at week 4 post exposure to air is shown by representative three-dimensional immunofluorescence staining taken by LSM. xy-projection is shown on top panel and xz-projection is shown on lower panel. Green: CFTR; Red: actin; blue: DAPI (nucleus). The micrographs presented are representative of data generated from 2 to 3 patients/donors

We characterized further the nasal CF ALI cultures by measuring the CFTR transcript levels in CF compare to control ALI cultures. We obtained similar CFTR mRNA levels in both cultures (Fig. 5a). Of note, the CFTR levels of the 5 CF ALI cultures tested where originating from CF patients with a F508del/ F508del genotype. Several in vitro studies support the idea of dysregulated inflammatory response in CF airway cells. In this context, IL-8 and IL-6 are widely used to assess the inflammatory state in CF cells and to measure the inflammatory response of CF cells upon infection [15, 18, 23]. Also, primary nasal epithelial cells produce significant levels of those cytokine under baseline condition [14, 24]. Thus, we measured the baseline levels of IL-6 and IL-8 cytokines produced basolateraly by CF in comparison to control ALI cultures. We didn’t found any significant differences between control and CF ALI cultures in terms of IL-6 (Fig. 5b), and IL-8 (Fig. 5c) baseline production. Finally, we assessed CFTR protein expression levels by immunocytochemistry and confocal imaging in CF nasal (Fig. 5d) in comparison to control (Fig. 5e). Since bronchial cells are the gold standard in the field, we used bronchial healthy ALI cultures as a reference (Fig. 5f). Of note the representative micrographs of CF nasal ALI cultures presented in Fig. 5d/g were originating from patients with a F508del/F508del genotype. We didn’t observe a notable difference in the CFTR signal/level between the different cultures tested (Fig. 5g-i).

## Discussion

The epithelium of the human respiratory tract provides the entry point and first line of defense against invading pathogens, particles and environmental pollutants [25]. CF airway epithelial cells have a dysfunction of several aspects of the innate immune response [26]. Thus, to investigate the role of the initiation of an appropriate immune response through release of soluble factors or direct cell-cell interaction, it is of particular importance to use a cell culture model that represents closely the in vivo situation with differentiation into ciliated, mucus-producing and basal cells.

Here we describe the establishment and characterization of fully differentiated primary pediatric CF airway epithelial cells grown at the ALI. Nasal epithelial cells are easily obtainable from pediatric patients and yield successful differentiated ALI cultures [11]. We showed that the epithelium of our differentiated CF cultures was pseudostratified with functional ciliated cells interspersed among mucus-secreting cells and also basal cells present, which largely mimics the airway epithelium in vivo*.* Further the epithelium was shown to be tight as demonstrated by the presence of tight junctional proteins (ZO-1) and an appropriate TEER. The ciliated cells showed an intact ciliary beat and the mucus-producing cells were functional in secreting mucus.

To date, fully differentiated CF airway epithelial cell cultures are not yet widely used and only few publications showed a partial characterization of those cultures. In the study of Sajjan et al., differentiated CF ALI cultures were characterized in terms of morphology, cytokine and mucin expression and TEER. The authors demonstrated the presence of ciliated and goblet cells and showed that ALI cultures of CF cells were functional for the production of IL-8 and mucin. Further, the epithelium was found to be tight characterized by an expression of the tight junction protein ZO-1. However, the group did not assess the functionality of the cilia such as beat frequency or assessment of coordinated beating. Moreover, the presence of basal cells in the differentiated epithelium was not investigated [4]. Wiszniewski et al. generated long-term CF ALI cultures on permeable inserts with a feeder layer of fibroblasts on their undersurface. The polarized CF cultures demonstrated the presence of basal, ciliated and mucus-secreting cells as well as the presence of the tight junction protein occludin on the apical cell membranes indicative of barrier function. Further, the characteristic elevated sodium transport, absence of cAMP-dependent transport of chloride and the dehydrated airway surface liquid compared to control ALI cultures have been shown. However, the drawback of this ALI cell culture method is that a biopsy is required to obtain both, patients fibroblast and airway epithelial cells [5]. Finally, de Courcey et al. established nasal CF ALI cultures and focused their evaluation on electrophysiological properties and partially evaluated the cellular content and IL-8 levels of the cultures [6].

The evaluation of CBF is a reliable method to assess the physiological status of the respiratory epithelium and of clinical importance in CF since the ciliary beat is a pivotal component of the mucociliary escalator, which is involved in pulmonary CF disease [21]. In our study, we therefore added the important aspect of ciliary beat to the CF ALI characterization by recording the CBF. We found a CBF of 9.67 Hz in the fully differentiated CF airway epithelial cell cultures. A recent study by Nair et al. showed a slightly higher baseline ciliary beat frequency of 12.9 Hz in differentiated CF ALI cultures [27]. Further in our study, a significant increase in tightness of the epithelium was observed in CF ALI compared to submerged cultures already at one-day post exposure to air. This suggests a rapid formation of tight junctions upon growing the cell culture at air.

Interestingly, it has for example been demonstrated that differentiated cell cultures are more resistant to rhinovirus (RV) infection, a relevant pathogen in CF [28, 29], than undifferentiated monolayer cell cultures, which resemble basal cells of the native epithelium [30]. Jakiela et al. [31] have shown that the basal cells of the differentiated epithelium are more susceptible to RV infection than the suprabasal cells proposing that differentiation is associated with resistance to RV infection. Thus, in conditions of damaged epithelium such as for asthma or CF, the susceptibility to infection might be increased and could contribute to lung disease. Therefore, a CF cell culture system with fully differentiated airway epithelial cells with the main cell types present and the possibility for co-culture with other cell types present in the respiratory tract such as neutrophils or alveolar macrophages is highly desirable.

In line with the observation from other investigators, we didn’t measure any significant difference in the levels of CFTR mRNA and/or protein in CF cultures generated from CF patients with the F508del/F508del genotype in comparison to control ALI cultures [32–34]. Also, there was no difference in baseline IL-6 and IL-8 levels in CF and control ALI cultures fully differentiated [18, 35, 36].

While airway epithelial nasal cultures are increasingly used as a surrogate to bronchial cells [12–14], there is evidence that differences between the upper and lower airway epithelium exist such at the level of gene expression and cytokine production [37–39]. When performing nasal cell-based studies, these limitations should be taken into account for the proper experimental design and the interpretation of the results.

In order to increase the flexibility of our method, cells cryopreservation after the initial phase of expansion under submerged conditions could be done. While we didn’t analyze this option, many investigators are using cryoconserved primary epithelial cells for the generation of ALI cultures, suggesting the possibility of cell storage for the subsequent generation of nasal CF ALI cultures [18].

## Conclusions

We successfully established fully differentiated pediatric CF nasal epithelial cells by nasal cytology brush sampling and characterized the cultures in terms of cellular content, ultrastructure, morphology and functionality. Our pediatric nasal CF ALI culture model mimicking the native human CF respiratory epithelium, is easy to establish/handle and could be used as surrogate of the pediatric bronchial epithelium and of animal-based approaches for basic CF studies.

## Additional files

Additional file 1: Macroscopic and microscopic view of mucus secretion by differentiated pediatric cystic fibrosis cell cultures grown at the air-liquid interface. (DOCX 3118 kb)

Additional file 2: Recording of ciliary beat by differentiated pediatric cystic fibrosis cell cultures grown at the air-liquid interface. (DOCX 136 kb)
