# Supplementary material for: Characterization of pediatric cystic fibrosis airway epithelial cell cultures at the air-liquid interface obtained by non-invasive nasal cytology brush sampling
Source: Respir Res. 2017 Dec 28;18:215. doi: 10.1186/s12931-017-0706-7 (PMC5745630; doi:10.1186/s12931-017-0706-7)
Supplement: Supplementary file 1 — Macroscopic and microscopic view of mucus secretion by differentiated pediatric cystic fibrosis cell cultures grown at the air-liquid interface. (DOCX 3118 kb) [file 12931_2017_706_MOESM1_ESM.docx]

**ADDITIONAL FILE 1**

**Characterization of cystic fibrosis airway epithelial cell cultures at the air-liquid interface obtained by non-invasive nasal cytology brush sampling**

Aline Schögler, Fabian Blank, Melanie Brügger, Seraina Beyeler, Stefan A. Tschanz, Nicolas Regamey, Carmen Casaulta, Thomas Geiser, and Marco P. Alves

| **A** | **B** |
| --- | --- |
| **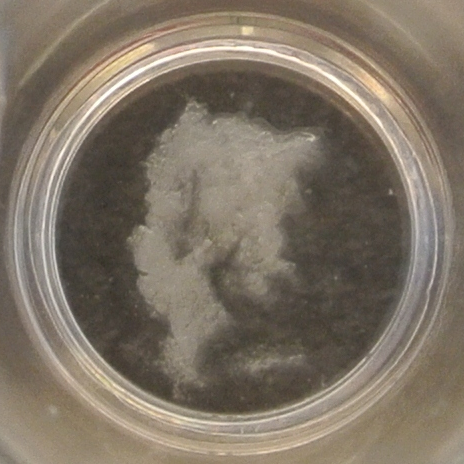** | **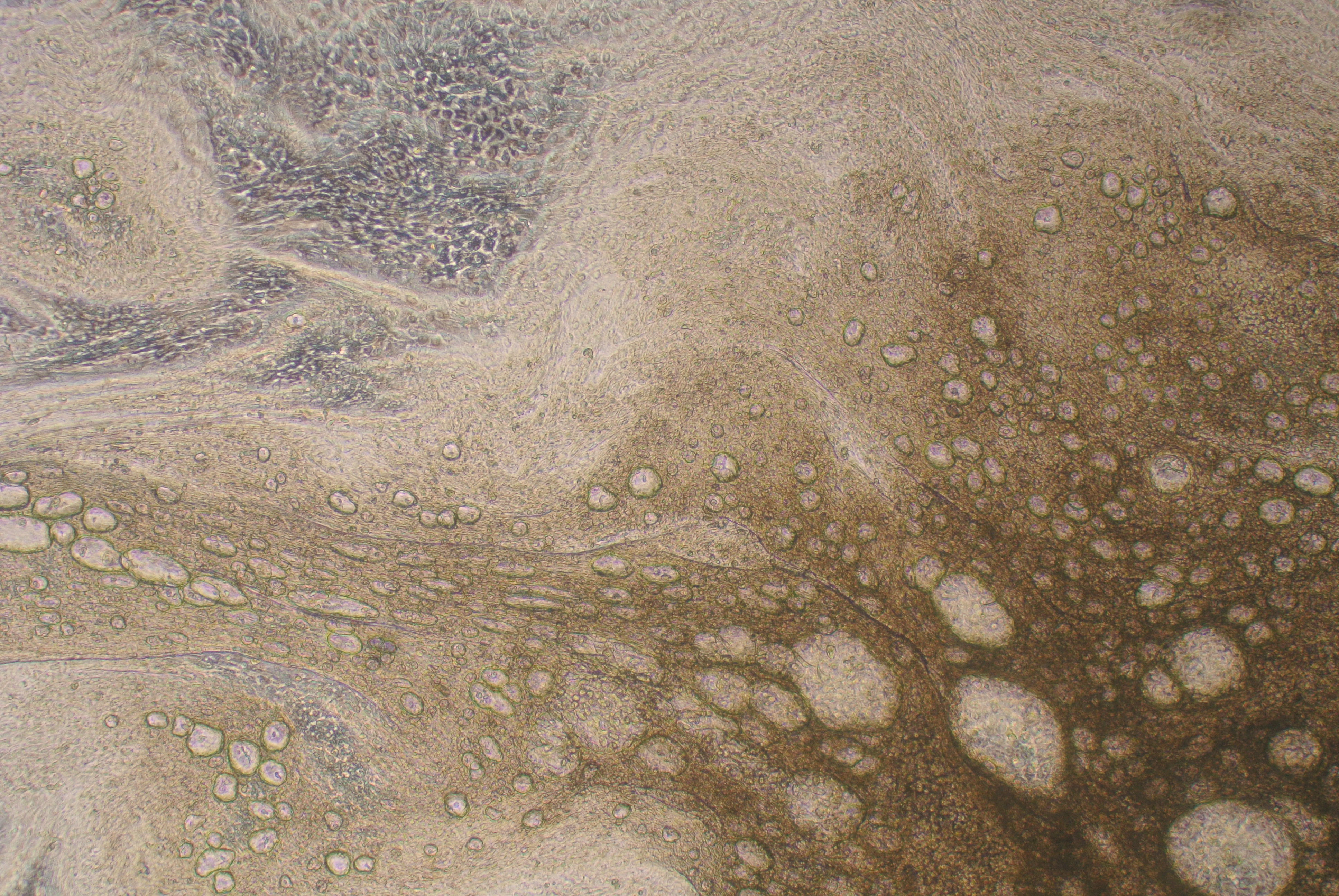** |
| **Macroscopic and microscopic view of mucus secretion by differentiated cystic fibrosis cell cultures grown at the air-liquid interface.** Representative pictures of mucus production of differentiated cystic fibrosis nasal epithelial cell cultures on a macroscopic view (A) and mucus production observed under the light microscope (4X magnification) (B) taken at week 2-3 post exposure to air. Photographs have been taken on a macroscopic scale with a conventional digital camera (Nikon, Switzerland) and on the microscopic scale with the same camera connected to the light microscope (Nikon, Switzerland) at a 4X magnification. | |
