# Supplementary material for: Characterization of pediatric cystic fibrosis airway epithelial cell cultures at the air-liquid interface obtained by non-invasive nasal cytology brush sampling
Source: Respir Res. 2017 Dec 28;18:215. doi: 10.1186/s12931-017-0706-7 (PMC5745630; doi:10.1186/s12931-017-0706-7)
Supplement: Supplementary file 2 — Recording of ciliary beat by differentiated pediatric cystic fibrosis cell cultures grown at the air-liquid interface. (DOCX 136 kb) [file 12931_2017_706_MOESM2_ESM.docx]

**ADDITIONAL FILE 2**

**Characterization of cystic fibrosis airway epithelial cell cultures at the air-liquid interface obtained by non-invasive nasal cytology brush sampling**

Aline Schögler, Fabian Blank, Melanie Brügger, Seraina Beyeler, Stefan A. Tschanz, Nicolas Regamey, Carmen Casaulta, Thomas Geiser, and Marco P. Alves

| **A** | **B** |
| --- | --- |
| **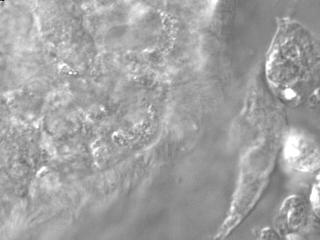** | **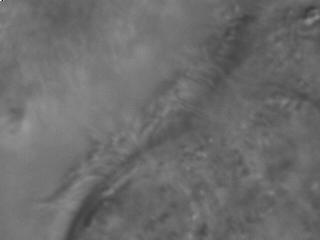** |
| **Recording of ciliary beat by differentiated cystic fibrosis cell cultures grown at the air-liquid interface.** Representative movies of ciliary beating and mucociliary transport of cell debris of well-differentiated cystic fibrosis nasal epithelial cell cultures (A, B). Image series of beating cilia were taken serially at 300 frames per second at a 40X magnification (as described in the main manucsript) and played at 50 frames per second. | |
